# Short-term Effects of Catheter Pressure and Time Control in Vacuum Aspiration Abortion for Early High-risk Pregnancies

**Published:** 2017-05

**Authors:** Lingna SUN, Yan Yu, Xiaoxia QI

**Affiliations:** Dept. of Gynecology, Shandong Provincial Maternity & Child-care Hospital, Jinan, Shandong, 250014, China

**Keywords:** Vacuum aspiration, High-risk early pregnancy, Short-term effect

## Abstract

**Background:**

We aimed to evaluate an approach to induced abortions during early pregnancies that controls the suction pressure and restricts the duration of the procedure.

**Methods:**

Three hundred patients programmed for induced early pregnancy abortions, hospitalized in the Shandong Provincial Maternity & Child-care Hospital from October 2013 to October 2015, were enrolled. Patients were randomly assigned to either research or control group. In the research group, operation pressure was controlled at 400 mmHg and operation time in the uterine cavity was kept at less than 75 s. In the control group, pressure ranged from 400–500 mmHg. Clinical variables were recorded for each patient until the fourth month after surgery, correlation and multivariate analyses were carried.

**Results:**

Compared with control group, anesthesia and intervention durations and the suction pressure were significantly lower and the endometrial thickness of the first late follicular phase after operation was significantly larger in the research group (*P*<0.05). In the first postoperative month, the number of patients who reported menstruation flow decreased by more than 1/3 of its normal volume was significantly lower than that in the control group (*P*<0.05). In the third postoperative month, the thickness of the late follicular phase endometrium was significantly larger than that in the control group (*P*<0.001). The mean intraoperative pressure and intrauterine operation duration both influenced the endometrial thickness of follicular phase.

**Conclusion:**

Controlling the suction pressure and time for vacuum aspiration abortions during early pregnancies can reduce the incidence of intrauterine adhesions and better protect the endometrium.

## Introduction

There are about 40 million to 60 million documented abortions annually worldwide, which means about 26% of the global pregnancies ends up in abortion (1). In China, around 13 million abortions are induced annually (2), of which 49.7% are in nonporous women, and 47.5% in women under the age of 25 years (3). Among women suffering from miscarriage, 55.9% have had a prior induced abortion; with 13.5% having had more than 3 previous abortions (3). The risk of intrauterine adhesions and infertility rises with the number of abortion procedures conducted on a woman. Vacuum aspiration is a surgical approach for terminating pregnancy; it is suitable for pregnancies within the first 10 weeks. A cannula is inserted into the uterine cavity and negative pressure is applied to pull out all embryotic tissues, therefore terminating the pregnancy.

This is a report on technical improvements to the procedure, implemented in our hospital, that reduce the endometrium trauma and the incidence of intrauterine adhesions.

## Materials and Methods

### Research subjects

Overall, 300 patients with early high-risk pregnancies admitted to Shandong Provincial Maternity & Child-care Hospital during the period running from October 2013 to October 2015 were enrolled in the study. All the patients were followed up for 4 months.

The inclusion criteria included an age in the range from 18–40 years, good physical condition, no history of oral mifepristone treatment, no contraindications for painless abortion procedure, presence of early high-risk pregnancy conditions (4) (that is, early pregnancy with history of more than 2 abortions per year or more than 3 abortions in total). The exclusion criteria included a gestational sac diameter greater than 5 cm or evidence of embryonic growth arrest on B-ultrasound images, and the unfeasibility to follow patients for 3 months after the operation.

The patients were randomly assigned to either a research group or a control group; each group had 150 cases. There were no significant differences between the two groups in terms of the age of patients or the size of gestational sacs (*P*>0.05) (Table 1).

**Table 1:** Clinical features of two groups of patients

**Group**	Cases	Age(yr)	Weight(kg)	Gestation age(day)	Gestational sac diameter(mm)	Gravidity(time)	Parity(time)
**Study**	150	27.9 ± 3.8	52.1 ± 4.1	60.5 ± 11.5	39.5 ± 9.6	2.8 ± 1.7	0.7 ± 0.7
**Control**	150	28.7 ± 3.6	51.8 ± 3.9	59.4 ± 12.5	40.8 ± 10.7	3.0 ± 1.8	0.8 ± 0.5

The Ethics Committee of Shandong Provincial Maternity & Child-care Hospital approved this study and all patients signed informed consent forms.

### Diagnosis and treatment process

The two groups of patients underwent a vacuum aspiration procedure according to the technical requirements. Specialists carried out the postoperative follow-ups. During the follow ups postoperative bleeding duration, time of restored menstruation, flow amount and duration were recorded. Additionally, endometrial thickness was detected by ultrasonography, and the presence of uterine adhesions detected by hysteroscopy, 4 months after the operation.

1) If the menstrual cycle recovered within 42 days after the operation, the return visit was planned in the first late follicular phase. 2) If menstruation was not present after 42 days, the return visit was done at the 42nd day and hysteroscopy was conducted to record intrauterine and cervical adhesions. 3) All patients returned for a visit three months after the operation, during the late menstrual follicular phase. 4) For patients with postoperative amenorrhea, those with reduced menstrual flow (less than ½ the normal amount), or those with a B-ultrasound showing endometrial thickness <0.5 cm at the first or the third postoperative month, a hysteroscopic screening was conducted at the fourth month after operation and intrauterine and cervical adhesions were recorded.

### Preoperative Vacuum aspiration preparation

Preoperative routine examinations for patients included gynecological examination, leucorrhea routine screening, blood lab results and ECG, 7–10 weeks of intrauterine pregnancy were diagnosed by B ultrasound, all tests gave normal results. The patients in the two groups were given mifepristone (35 mg/tablet, Hubei Gedianrenfu Pharmaceutical, approval number: H20040365) 24 hours before the procedure, b.i.d. with a 12-h interval, and misoprostol tablets 600μg orally, the next morning given before a meal (200 μg/tablet, Cytotec, approval number: imported drug registration H20100186). Next, 2 hours later, the conventional painless vacuum aspiration was performed; the procedure was conducted under intravenous general anesthesia provided by an anesthesiologist, propofol combined with fentanyl (Yichang Renfu Pharmaceutical, 3 ~ 6 mg/kg/h).

### Vacuum aspiration operation

The actual aspiration operations were carried out with differences in the two groups of patients performed by the same physician. For the research group a suction head 7 or 8 was chosen according to the size of the gestational sac. A first suction round was carried out pointing the suction catheter towards the gestational sac, under a pressure of ≤400 mmHg. The second round had the catheter scraping the uterine cavity carefully with a pressure of ≤200 mmHg. The third round was performed to check around the uterine cavity with suction head 6 again, under a pressure of 100 mmHg. The force applied against the uterine cavity was always recoiled at any feeling of roughness in the cavity, to avoid damage to the basal membrane of the endometrium. The whole operation time in the cavity was less than 75s, the anesthesia and intrauterine operation durations, and the pressures applied with the catheters were recorded. For the control group, the surgical standard pressure was maintained at 400–500 mmHg, the uterine cavity operation time was not restricted, the procedure was terminated by the surgeon according to her own judgment by feeling uterine contraction and uterine wall roughness. The anesthesia duration, intrauterine operation time and mean pressure were recorded.

### Criteria for judging menstrual amount and late follicular phase endometrium thickness

The normal menstrual amount per cycle is thought to be around 30 ml. However, there are no practical means of accurately measuring the menstrual amounts in patients. Therefore, the patients were asked to compare the recent total menstrual amount with the amounts of prior cycles. Those patients who claimed to have a reduction of more than 1/3 of the prior quantities were considered to have decreased menstrual amounts. Alternatively, those who claimed an increase of more than 1/3 were considered to have increased menstrual amounts. Any perceived significant changes in the menstrual amount were considered abnormal (5).

The endometrium’s thickness is affected by the cyclical changes in estrogen and progesterone levels. The early follicular endometrium shows a trilaminar pattern; the middle and late stage endometriums show a significant contrast between the functional and the basal layers, therefore the detection of the thickness was performed during the late follicular phase using a transvaginal ultrasound machine.

### Hysteroscopy

According to the European Society for Gynecological Endoscopy (ESGE), the intrauterine adhesions observed through hysteroscopy can be divided into Classes I through V. Class I includes multiple filmy adhesions in the uterine cavity, with both sides of the cornua uteri and the opening of the fallopian tubes normal. Class II consists of dense fibrous adhesions between the anterior and the posterior walls of the uterus, both cornua uteri and the opening of the fallopian tubes are still visible. In class III the stringy, fibrous adhesions cause occlusion of part of the uterus cavity and one side of the cornua uteri. Class IV includes fibrous adhesions that occlude part of the uterine cavity and both sides of the cornua uteri. In the yet more serious class V the adhesion scarring causes extensive deformation and stenosis of the uterine cavity, and the walls are completely shielded due to the adhesions. For this study, class I was considered as mild, II–III were considered as moderate, and IV–V were considered severe (6,7).

### Statistical analysis

All data were analyzed using the SPSS.21.0 software (IBM, NYC, USA). The measurement data were expressed as mean ± standard deviation. The *t* test was used to compare the mean value between groups and the χ^2^ test was used to compare the enumerative data between groups. For the univariate analysis the Spearman method was used to analyze the correlations in the data; the multivariate analysis used the Logistic method. A *P*<0.05 was considered as statistically significant.

## Results

### Comparison of anesthesia and operation durations, catheter pressure and blood loss averages (X ± s)

We compared the mean values for the variables between the research and the control group. The results showed that compared with the control group, the anesthesia and operation durations and the catheter pressures were significantly decreased in the research group, (*P*<0.05). There was no significant difference between the two groups in regards to the intraoperative blood loss (*P*>0.05) (Table 2).

**Table 2:** Clinical conditions of patients during operation

**Group**	Cases	Anesthesia time(min, x ± s)		Operation time(min, x ± s)		Pressure(mmHg, x ± s)		Blood loss(ml, x ± s)
		X̄ ± s	95%CI	X̄ ± s	95%CI	X̄ ± s	95%CI	X̄ ± s
**Research**	150	5.81 ± 1.51	2.85∼8.77	1.19 ± 0.22	0.76∼0.51	276.8 ± 68.7	142.15∼411.45	18.46 ± 9.87
**Control**	150	7.35 ± 1.42	4.57∼10.13	2.18 ± 0.48	1.24∼6.32	340.7 ± 58.9	225.3∼456.1	19.78 ± 8.90

### Uterine recovery after operation

We followed up the two groups of patients during visits after the initial procedure. No significant differences were found between the groups concerning variables such as postoperative bleeding duration, the time until menstruation restoration, presence and severity of cervical adhesions, or any other complications (*P*>0.05) (Table 3).

**Table 3:** Postoperative uterine recovery

**Group**	Cases	Postoperative bleeding time (days)	Time of re-menstruation (days)	Amenorrhea (cases)	Cervical adhesion (cases)	Intrauterine residual (cases)
**Observation**	150	6.7 ± 2.6	37.3 ± 6.6	3	0	0
**Control**	150	6.1 ± 2.8	38.6 ± 4.7	5	2	0

### Evaluation of endometrial thickness and menstrual flow amounts

The average endometrial thicknesses of the subjects in the two groups were examined one month and three months after the operation. It was observed that, in the research group, the endometrial thickness average during the late follicular phase one month after operation was significantly larger than that in the control group, (*P*<0.001). The number patients complaining about menstrual flow amounts having decreased by 1/3, one month after the operation, were significantly lower in the research group, (*P*<0.05). The average endometrial thickness during the late follicular phase, three months after operation, was significantly larger in the research group (*P*0.001). However, for the number of patients complaining about menstrual flow being reduced by ½, no statistically significant differences were found between the two groups (Table 4).

**Table 4:** Menstrual flow and endometrial thickness one and three months after operation

**Group**	Cases	1 month after operation		3 months after operation	
		Endometrial thickness, late follicular phase (mm)	Perceived menstrual flow decrease (case)	Endometrial thickness, late follicular phase (mm)	Perceived menstrual flow decrease (case)
**Research**	150	6.4 ± 0.6	21	7.8 ± 1.3	14
**Control**	150	5.1 ± 0.8	35	6.5 ± 0.9	15

### Hysteroscopy results four months after operation

Four months after operation, hysteroscopies were performed in all patients. There were no significant differences between the two groups in regards to class I–V adhesions (*P*>0.05) (Table 5).

**Table 5:** Hysteroscopy results (4 months after operation)

**Group**	Cases	Cases of hysteroscopy	Normal cavity	I – II Intrauterine adhesion^b^	III Intrauterine adhesion^b^	IV Intrauterine adhesion^b^
**Research**	150	35	10	25	0	0
**Control**	150	50	12	36	2	0

### Multivariate correlative analysis of endometrial thickness three months after operation

Based on the above results, we tried to find out the influencing factors for different prognosis of the two groups of patients. We took intrauterine operation time, mean catheter pressure, endometrial thickness in late follicular phase one month after operation, perceived menstrual flow decrease as variables, and took the endometrial thickness during the follicular phase three months after operation as a constant, in order to conduct a multivariate correlative analysis. The result showed that mean catheter pressure and intrauterine operation duration are both influencing factors for the endometrial thickness during the late follicular phase three months after operation, (*P*<0.05) (Table 6).

**Table 6:** Multivariate correlative analysis

**Variable**	β	SE	β’	OR (95%CI)	
				Upper limit	Lower limit
**Age (yr)**	0.431	0.13	0.564	0.61	0.16
**Size of gestational sac**	0.721	0.12	0.442	0.75	0.29
**Intrauterine operation time**	0.278	0.32	0.372	0.79	0.11
**Intrauterine operation pressure**	0.387	0.31	0.161	0.65	0.17
**Anesthesia duration**	0.749	0.28	0.961	0.82	0.31
**Postoperative bleeding time**	0.633	0.18	0.378	0.55	0.17
**Time to menstruation restoration**	0.762	0.21	0.478	0.78	0.23
**Cervical adhesion**	0.273	0.33	0.372	0.65	0.13
**Residues left in Intrauterine cavity**	0.283	0.18	0.135	0.54	0.22
**Endometrial thickness during late follicular phase one month after operation**	0.128	0.15	0.367	0.58	0.18
**Menstrual flow decrease one month after operation**	0.327	0.17	0.392	0.78	0.12
**Menstrual flow decrease three months after operation**	0.548	0.18	0.144	0.56	0.19
**Nursing time after operation**	0.673	0.14	0.233	0.37	0.10

## Discussion

In China, induced abortions are legal as a remedial measure for contraceptive failure. The number of induced abortions in China accounts for 1/4 of the global number, and about 1/3 of the induced abortions are recurrent abortions (8). Damage to the uterine cavity and cervical mucosa during abortion operations can be due to several factors including the excessive scraping of the intrauterine walls, an overly high negative pressure during suction, an inappropriate cervical expansion procedure, a curette not matching the time of pregnancy, the repeated introduction of a suction head or curette into the uterine cavity, and the application of negative pressure while entering through the cervix (9). Patients undergoing painless induced abortion procedures have poor uterus contraction and extensive bleeding under anesthesia, which can lead to excessive curettage and endometrial damage by a physician who mistakes these as signs of an insufficiently cleaned surface, increasing the risk for intrauterine adhesions (7). According to gynecological infertility statistics, 30%–50% of infertilities are caused by intrauterine adhesions (2). For recurrent abortion patients, the repeated scratching of the intrauterine walls leads to the endometrial damage, causing higher intrauterine infection and intrauterine adhesion rates. Researchers have linked the history of multiple dilation and curettage procedures in multiple abortions to an increased incidence of intrauterine adhesions (9). Others have reported that the percentage of intrauterine adhesions in induced abortion patients reaches 13.21% (10), much higher than the 3% among general population.

Previous evidence (11) call for the choice of the pressure applied to the rubber catheter to be between 400 and 500 mmHg according to the gestational age and uterine size. One or two rounds of suction clockwise should be stopped when the uterine cavity feels rough, suggesting the tissues are cleaned; and then a gentle curettage should be used for the uterine fundus and the cornua uteri, if necessary. The Chinese Medical Association promulgating the " Clinical skills practice oligogenics fascicle " in 2004 (4) recommended instead to first direct the suction catheter to the location of the embryo under 400–500 mmHg open negative pressure, and then rotate the suction head clockwise or counterclockwise, moving up and down until feeling the uterine contractions and wall’s roughness. Then decrease the negative pressure to 200–300 mmHg, and apply one or two suction rounds again before taking the cannula out. If necessary, the use of a small curette to gently curettage the fundus and cornua is advised. The Chinese Medical Association has not yet revised its 2004 clinical practice guidelines. These days, more informed patients are more concerned about possible complications of the procedure such as uterine perforation, residual tissues and bleeding. However, the effects of long time high-pressure intrauterine operations on the patient's postoperative endometrium repair and conception capabilities are mostly overlooked (5).

In this study, we tested an approach where the actual operating pressure in the uterine cavity was decreased, and the duration of the procedure was restricted in the research group. The results showed that the intraoperative blood loss, postoperative bleeding time, menstruation restoration time, and other factors were not significantly different between the two groups (*P*>0.05). However, in the research group, the operation and the anesthesia durations were significantly reduced (*P*<0.001). More importantly, the endometrial thickness in the late follicular phase one and three months after operation were significantly higher than those in the control group (*P*<0.001). Taken together, we suggest that appropriate control on pressure and intrauterine operation duration cannot only reduce the risks associated with the length of surgery and anesthesia, but also have a positive effect on the protection of the uterine cavity and cervix.

In terms of endometrial repairing, although the two groups did not show significant differences in postoperative follow ups, the control group had a higher absolute value of complications (5 cases of amenorrhea, 2 cases of cervical adhesions); while the research group had only 3 cases of amenorrhea and no cervical adhesions. In addition, though there were no significant differences in the number of mild adhesions between the two groups, no severe adhesions were found in the research group. The lack of significant differences between the groups might be because the hysteroscopies were performed too early, or due to the small samples in the study. The degree of intrauterine adhesions increases with age and the presence of menopause; and the prolonged presence of intrauterine adhesions may lead to fibrosis or scarring, causing even permanent damage to the endometrium (12). We used the intrauterine thickness during the late follicular phase in the third month after the operation as an independent variable to analyze the impact of various other variables. We found that the intrauterine operation duration and the suction pressure were important factors.

Therefore, the results here seem to indicate that keeping the negative suction pressure and the operation duration time to a minimum, may significantly improve the degree of intrauterine adhesions, the postoperative endometrium thickness, the menstrual amounts, etc. The timely follow-ups and early interventions play also a significant role in the protection of the endometrium of the patients.

## Conclusion

An improved suction abortion procedure with reduced intrauterine operation pressures and operating duration can lead to better clinical results, reducing the trauma to the endometrium, lowering the incidence of intrauterine adhesions, and therefore better protecting the fertility capabilities of women undergoing early pregnancy induced abortions.

## Ethical considerations

Ethical issues (Including plagiarism, informed consent, misconduct, data fabrication and/or falsification, double publication and/or submission, redundancy, etc.) have been completely observed by the authors.

## References

[1] Henshaw SK Singh S Haas T (1999). The incidence of abortion worldwide. Int Fam Plann Persp, 25(Suppl):S30–8. 14627053

[2] Qian X Tang S Garner P (2004). Unintended pregnancy and induced abortion among unmarried women in China: a systematic review. BMC Health Serv Res, 4:1. 14736336 10.1186/1472-6963-4-1 PMC333425

[3] Cheng L (2006). Medical abortion in early pregnancy: experience in China. Contraception, 74(1):61–65. 16781263 10.1016/j.contraception.2006.03.004

[4] Guiahi M Davis A Society of Family Planning (2012). First-trimester abortion in women with medical conditions: release date October 2012 SFP guideline #20122. Contraception, 86(6):622–30. 23039921 10.1016/j.contraception.2012.09.001

[5] Norman WV Bergunder J Eccles L (2011). Accuracy of gestational age estimated by menstrual dating in women seeking abortion beyond nine weeks. J Obstet Gynaecol Can, 33(3):252–257. 21453565 10.1016/s1701-2163(16)34826-5

[6] Abdalla HI Brooks AA Johnson MR Kirkland A Thomas A Studd JW (1994). Endometrial thickness: a predictor of implantation in ovum recipients? Hum Reprod, 9(2):363–5. 8027298 10.1093/oxfordjournals.humrep.a138509

[7] Hooker AB Lemmers M Thurkow AL (2014). Systematic review and meta-analysis of intrauterine adhesions after miscarriage: prevalence, risk factors and long-term reproductive outcome. Hum Reprod Update, 20(2):262–78. 24082042 10.1093/humupd/dmt045

[8] Cheng Y Gno X Li Y Li S Qu A Kang B (2004). Repeat induced abortions and contraceptive practices among unmarried young women seeking an abortion in China. Int J Gynaecol Obstet, 87(2):199–202. 15491580 10.1016/j.ijgo.2004.06.010

[9] Chen Y Chang Y Yao S (2013). Role of angiogenesis in endometrial repair of patients with severe intrauterine adhesion. Int J Clin Exp Pathol, 6(7):1343–1350. 23826415 PMC3693199

[10] Friedler S Margalioth EJ Kafka I Yaffe H (1993). Incidence of post-abortion intrauterine adhesions evaluated by hysteroscopy - A prospective study. Hum Reprod, 8(3):442–444. 8473464 10.1093/oxfordjournals.humrep.a138068

[11] Römer T (1994). Post-abortion-hysteroscopy--a method for early diagnosis of congenital and acquired intrauterine causes of abortions. Eur J Obstet Gynecol Reprod Biol, 57(3):171–3. 7713291 10.1016/0028-2243(94)90295-x

[12] Li L Nai M Gao G Wang L (2016). [Comparison among measures to prevent intrauterine adhesions after artificial abortion]. Zhong Nan Da Xue Xue Bao Yi Xue Ban, 41(9):975–978 (In Chinese). 27640797 10.11817/j.issn.1672-7347.2016.09.013

